# Parasite spread at the domestic animal - wildlife interface: anthropogenic habitat use, phylogeny and body mass drive risk of cat and dog flea (*Ctenocephalides* spp.) infestation in wild mammals

**DOI:** 10.1186/s13071-017-2564-z

**Published:** 2018-01-08

**Authors:** Nicholas J. Clark, Jennifer M. Seddon, Jan Šlapeta, Konstans Wells

**Affiliations:** 1 School of Veterinary Science, University of Queensland, Gatton, QLD 4343 Australia; 2 Sydney School of Veterinary Science, Faculty of Science, University of Sydney, Sydney, NSW 2006 Australia; 3 Environmental Futures Research Institute, Griffith University, Nathan, QLD 4111 Australia

**Keywords:** *Ctenocephalides canis*, *Ctenocephalides felis*, Domestic animal - wildlife interface, Ecological fitting, Host specificity, Invasive species, Reservoir host, Spillover

## Abstract

**Background:**

Spillover of parasites at the domestic animal - wildlife interface is a pervasive threat to animal health. Cat and dog fleas (*Ctenocephalides felis* and *C. canis*) are among the world’s most invasive and economically important ectoparasites. Although both species are presumed to infest a diversity of host species across the globe, knowledge on their distributions in wildlife is poor. We built a global dataset of wild mammal host associations for cat and dog fleas, and used Bayesian hierarchical models to identify traits that predict wildlife infestation probability. We complemented this by calculating functional-phylogenetic host specificity to assess whether fleas are restricted to hosts with similar evolutionary histories, diet or habitat niches.

**Results:**

Over 130 wildlife species have been found to harbour cat fleas, representing nearly 20% of all mammal species sampled for fleas. Phylogenetic models indicate cat fleas are capable of infesting a broad diversity of wild mammal species through ecological fitting. Those that use anthropogenic habitats are at highest risk. Dog fleas, by contrast, have been recorded in 31 mammal species that are primarily restricted to certain phylogenetic clades, including canids, felids and murids. Both flea species are commonly reported infesting mammals that are feral (free-roaming cats and dogs) or introduced (red foxes, black rats and brown rats), suggesting the breakdown of barriers between wildlife and invasive reservoir species will increase spillover at the domestic animal - wildlife interface.

**Conclusions:**

Our empirical evidence shows that cat fleas are incredibly host-generalist, likely exhibiting a host range that is among the broadest of all ectoparasites. Reducing wild species’ contact rates with domestic animals across natural and anthropogenic habitats, together with mitigating impacts of invasive reservoir hosts, will be crucial for reducing invasive flea infestations in wild mammals.

**Electronic supplementary material:**

The online version of this article (10.1186/s13071-017-2564-z) contains supplementary material, which is available to authorized users.

## Background

Animals closely associated with humans can act as reservoir hosts that spread parasites to wildlife [1–3]. Spillover of parasites (i.e. the transmission of a parasite from one host species to another) between domestic and wild animals is an increasing threat to animal health, and understanding factors that drive this process is crucial [4–6]. Yet while conversion of natural habitat into production zones, habitat fragmentation and global urbanisation increase contact rates between domestic and wild animals [7, 8], patterns of parasite sharing at the domestic animal - wildlife interface are poorly resolved.

Cat fleas (*Ctenocephalides felis*) and related dog fleas (*C. canis*) are blood-feeding ectoparasites causing enormous grievances for pets worldwide [9–12]. Flea control relies on mass use of preventative drugs, equating to hundreds of dollars spent by owners each year [13]. In addition to pets, *C. felis* and *C. canis* are presumed to infest a diversity of wild species. Control of parasite spread and infestation-related morbidity are therefore multifaceted problems [14–17]. The potential for urban-wildlife parasite exchange represents a considerable One Health threat, especially since fleas can transmit harmful bacteria (some of them being zoonotic [18, 19]). Despite the pervasive risk for flea spillover between domestic and wild animals, there is a dearth of knowledge on *C. felis* and *C. canis* distributions among wildlife [10, 20, 21].

Predicting parasite spread requires an understanding of wildlife characteristics that enable host shifting [22]. The human-induced range expansion of domestic animals and other non-native species that act as viable hosts for fleas (including foxes, rabbits and rats [23–25]) has led to the encroachment of potential reservoir host species into almost all terrestrial environments [26–28]. Close proximity between natural and anthropogenic habitats might increase exposure to feral and domestic animals [29–31] and could be a key predictor of *C. felis* and *C. canis* infestation in wildlife. However, other host attributes, such as body mass, diet and phylogenetic ancestry, can be informative for predicting whether hosts share parasite species [3, 32]. These attributes may facilitate flea exchange, as factors regulating habitat use are important drivers of ectoparasite infestation [33–35].

How historical and ecological species traits facilitate or inhibit flea infestation is not known. Moreover, information on infestation rates in wildlife species is scattered throughout the literature. We use a systematic literature search and web scraping tools to build a global database of *C. felis* and *C. canis* infestations in wildlife species. Using Bayesian hierarchical models, we incorporate mammalian trait data to ask if extrinsic (habitat use, diet breadth) and intrinsic (phylogenetic ancestry, body mass) host attributes act as drivers of flea infestation risk. We use host specificity analyses and null models to assess whether fleas infest species that are more similar in their phylogenetic ancestry, habitat use or diet than expected by chance. If habitat use is a key driver of infestation risk, we expect that use of anthropogenic habitats will increase species’ infestation probability and that both flea species will infest hosts that exhibit more similar habitats than expected by chance.

## Results

### Introduced mammals as reservoir hosts for fleas at the domestic-animal wildlife interface

**Fig. 1:**
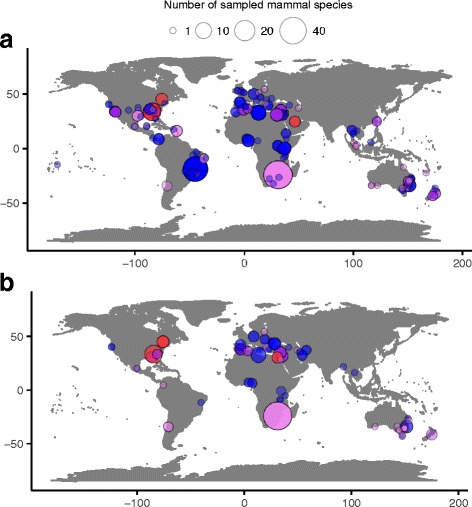
Geographical distributions of observed cat flea *Ctenocephalides felis* (**a**) and dog flea *C. canis* (**b**) infestation reports in free-roaming mammals around the globe. Sizes of points represent the number of mammal species sampled in each record. Colours correspond to the total number of feral host species observed to carry fleas at each location (blue = 0, purple = 1, magenta = 2, pink = 3, red = 4)

Both flea species infest wildlife on all continents apart from Antarctica (Fig. 1). In total, 138 (20%) out of 685 sampled wild mammal species harboured cat fleas (*Ctenocephalides felis*) and 31 (4%) harboured dog fleas (*C. canis*). Species most frequently reported to be associated with *C. felis* were all invasive mammals, including feral cats (26 out of 446 total flea-host-location observations; ranging all sampled continents), feral dogs (21 observations), red foxes (*Vulpes vulpes*; 19 observations), black rats (*Rattus rattus* species complex; 16 observations), brown rats (*Rattus norvegicus*; 14 records) and European rabbits (*Oryctolagus cuniculus*; 9 observations; Table 1). Likewise, *C. canis* was commonly reported infesting feral mammals, including red foxes (22 observations), feral dogs (12 observations), feral cats (8 observations), and black and brown rats (5 observations each; Table 1). From studies that included prevalence information, mean *C. felis* prevalence was highest in feral cats (mean 32.3%). *Ctenocephalides canis* prevalence was highest in red foxes (mean 3.5%; Table 1). While these observations may be biased by more intensive sampling of invasive mammals, especially if there is greater incentive to publish on invasive species, they suggest invasive mammals act as suitable reservoir hosts for cat and dog fleas.

**Table 1:** Sampling frequencies and prevalences of cat and dog fleas (*Ctenocephalides felis* and *C. canis*) in selected invasive host species. Note that prevalence information was not available from all studies included in the database

Host species	No. of studies that recorded prevalence (no. of countries)	No. of individuals included in prevalence calculation	Mean *C. felis* prevalence (range) (%)	Mean *C. canis* prevalence (range) (%)
*Felis catus* (feral cat)	27 (18)	2974	32.3 (0–100)	1.3 (0–34.8)
*Canis lupus* (feral dog)	19 (16)	1941	17.1 (0–92.1)	3.5 (0–30.1)
*Vulpes vulpes* (red fox)	9 (8)	2118	15.9 (0–100)	11.5 (0–100)
*Rattus rattus* (black rat)	7 (6)	1327	1.4 (0–10.1)	No prevalence information
*Rattus norvegicus* (brown rat)	4 (4)	1458	0.4 (0–1.0)	No prevalence information
*Oryctolagus cuniculus* (European rabbit)	1 (1)	8	100	No prevalence information

Among native species, *C. felis* was commonly reported in American opossums (Virginia oppossum *Didelphis virginianam*: 7 observations, and common oppossum *Didelphis marsupialis:* 6 observations), North American gray foxes (*Urocyon cinereoargenteus*: 5 observations), and Australian brushtail possums (*Trichosurus vulpecula*: 3 observations). For *C. canis*, commonly reported native species included Iberian lynx (*Lynx pardinus*: 4 observations), North American gray foxes (3 observations) and a variety of other wild carnivores (including the coyote *Canis latrans*, golden jackal *Canis aureus*, and common gennet *Genetta genetta*).

### Host phylogeny, body mass and anthropogenic habitat use drive parasite infestation risk

Host phylogeny explained considerable variation in *C. felis* infestation probability, accounting for 64.9% of variation (CI: 45.4–74.2%). *Ctenocephalides felis* infestation probability decreased with increasing host body mass (accounting for 41.2% of the remaining explained variation; CI: 1.2–79.7%), with a decrease of 1 kg in mean body mass equating to an increase of 0.6% in infestation probability (CI: 0.2–2.8%). This could either mean that large body size prevents infestation or, more likely, that larger mammals are less likely to overlap human habitats. As expected, anthropogenic habitat use was a strong positive predictor of *C. felis* infestation (accounting for 22.3% of remaining explained variation;CI: 1.6–54.9%), with odds of infestation for anthropogenic habitat-using species increasing by 256% compared to species that do not use anthropogenic habitats (CI: 125.9–687.8%). Credible intervals for all other coefficients included zero (Additional file 1: Figure S1).

For *C. canis*, infestation probability was linked to host phylogeny (19.2% of explained variation; CI: 6.4–33.5%) when accounting for a significant positive effect of total citations associated with the term ‘ectoparasite’ (6.3% of explained variation; CI: 0.4–28.5%). Similarly to *C. felis*, infestation probability for *C. canis* increased with decreasing host body mass (accounting for 77.4% of remaining explained variation; CI: 14.2–96.7%). Infestation probability for *C. canis* is predicted to increase by 2.7% (CI: 0.2–11.3%) with a decrease of 1 kg in host body mass. The use of anthropogenic habitats was weakly positive but non-significant for predicting *C. canis* infestation (regression coefficient CI: −0.34–2.87), suggesting that more data is needed to elucidate this possible pattern. Credible intervals for all other regression coefficients included zero (Additional file 1: Figure S1).

**Fig. 2:**
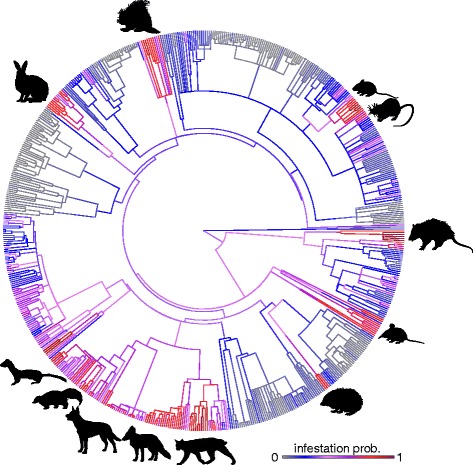
Cat flea (*Ctenocephalides felis*) infestation probability in wild mammals, mapped across a phylogeny of 639 sampled mammal species. Colours represent ancestral state mapping of predicted infestation probability, calculated by entering species’ attributes into fitted logistic regression equations (using posterior modes for regression coefficients and variable intercepts according to phylogenetic ancestry). Cooler blues indicate low infestation probability; warmer reds show high infestation probability. Key phylogenetic host groups (i.e. clades in which multiple species show above 0.7 infestation probability) are indicated with outline figures (clockwise from top: porcupines (Hystricidae); mice and rats (Muridae); possums and oppossums (Phalangeridae, Didelphidae); shrews (Soricidae); hedgehogs (Erinaceidae); felines (Felidae); foxes (genus *Vulpes*; Canidae); dogs (genus *Canis*; Canidae); skunks (Mephitidae); and weasels (Mustelidae). Images were sourced from http://www.supercoloring.com under a Creative Commons License (https://creativecommons.org/licenses/by/4.0/)

**Fig. 3:**
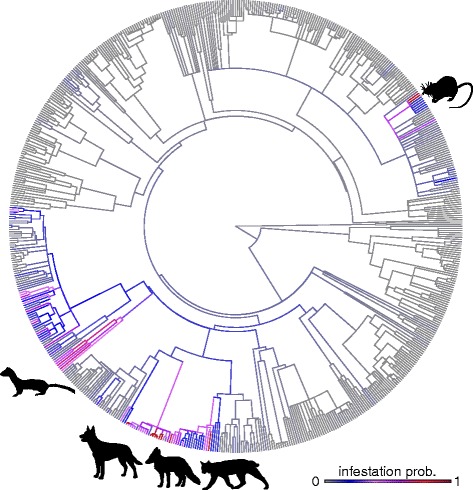
Dog flea (*Ctenocephalides canis*) infestation probability in wild mammals, mapped across a phylogeny of 639 sampled mammal species. Colours represent ancestral state mapping of the fitted infestation probability, calculated by entering species’ attributes into fitted logistic regression equations (using posterior modes for regression coefficients and variable intercepts according to phylogenetic ancestry). Cooler blues indicate low infestation probability; warmer reds show high infestation probability. Key phylogenetic host groups (i.e. clades in which multiple species show above 0.7 infestation probability) are indicated with outline figures (clockwise from top: rats (Muridae); felines (Felidae); foxes (genus *Vulpes*; Canidae); dogs (genus *Canis*; Canidae); and weasels (Mustelidae). Images were sourced from http://www.supercoloring.com under a Creative Commons License (https://creativecommons.org/licenses/by/4.0/)

Entering species’ ecological traits (from all sampled mammalian hosts for which we had phylogenetic data; *n* = 639) into equations from fitted regressions (using coefficient posterior modes and phylogenetic variable intercepts) revealed two key patterns. First, although *C. felis* infestation probability shows a phylogenetic signal (related species showing similar infestation risk), this parasite is predicted to infest a wide diversity of mammals covering the majority of clades along the sampled host phylogeny (Fig. 2). According to the model, species with particularly high risk of *C. felis* infestation include many canids, felids and murids, in addition to host species such as possums (Phalangeridae and Didelphidae), skunks (Mephitidae), shrews (Soricidae), weasels (Mustelidae) and old world porcupines (Hystricidae). Secondly, *C. canis* is predicted to infest a much lower diversity of species, with susceptible hosts primarily including wild canids, felids, murids and mustelids (Fig. 3).

### Dog fleas infest phylogenetically clustered mammalian host species

**Fig. 4:**
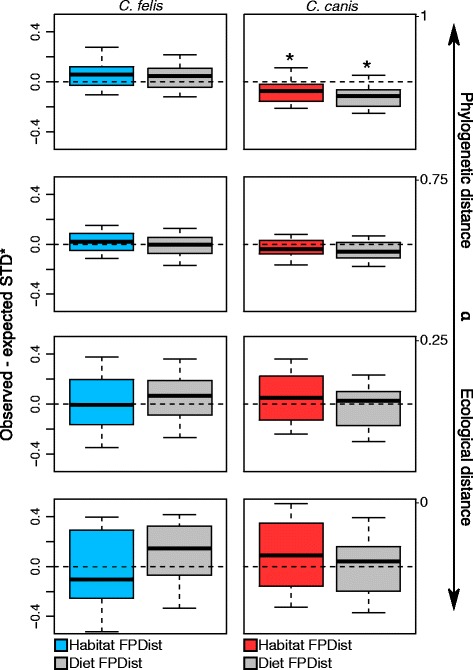
Differentials between observed and expected functional-phylogenetic host specificity (STD*) for dog fleas (*Ctenocephalides canis*; left panel) and cat fleas (*C. felis*; right panel) at varying α weights. Weighting values approaching 0 give more weight to host ecological distance, while values approaching 1 give higher weight to host phylogenetic distance. Negative differentials indicate infested hosts are more similar than expected by chance; positive values indicate infested hosts are more dissimilar than expected. Differentials were generated from 10,000 iterations, using a mammalian supertree [72] and either randomly sampled host habitat dendrograms (coloured boxes) or host diet niche dendrograms (grey boxes) in each iteration. Boxplots show differential medians (lines within boxes), and 2.5% and 97.5% quartiles (hinges) for individual parasites. Whiskers show minimum and maximum values. Asterisks (*) indicate significant differences from 0

For *C. canis*, host specificity intervals became significantly clustered as phylogenetic weight increased (*a* values approaching 1; Fig. 4), indicating infested hosts were more closely related than expected by chance. As ecological niche weight increased (*a* values approaching 0), intervals overlapped zero, suggesting *C. canis* hosts did not exhibit more similar habitat or diet niches than expected (Fig. 4). For *C. felis*, in contrast, host specificity intervals included zero for all *a* weighting values, suggesting infested hosts were not more closely related to each other nor did they exhibit more similar habitat or diet niches than expected by chance (Fig. 4).

## Discussion

Management strategies to mitigate parasite spillover require identifying host attributes that increase infestation risk [3, 36, 37]. We find that the use of anthropogenic habitats is a key driver of cat flea infestation risk. As habitat encroachment accelerates [8, 38], increased contact between wild mammals and human-associated reservoir hosts is likely to increase spillover of cat fleas to wildlife. While intrinsic host attributes such as phylogenetic ancestry and large body mass may ameliorate risk for some species, our findings suggest a large diversity of species are susceptible to cat flea infestation. In contrast, dog fleas are less widespread and more restricted to hosts with shared evolutionary histories. Future spillover of dog fleas, in turn, is expected to be more strongly confined to a few phylogenetic groups, reducing their overall spread compared to the more host-generalist cat fleas. Contact patterns between potential host species will not only depend on habitat overlap, but also on species-level behavioural and population-level demographic attributes [39]. Understanding within-population infestation dynamics and mitigating impacts of invasive reservoir hosts will therefore be crucial for reducing flea spillover at the domestic-wildlife interface.

Although it is often stated that cat and dog fleas are cosmopolitan parasites infesting a diversity of species [9, 10, 40], this is the first study to uncover the magnitude and geographic spread of their wildlife occurrences. In doing so, we provide tangible evidence that invasive species contribute to the spread of the most common parasites of pets in human households. Numerous feral mammal species were identified as important reservoir hosts for both cat and dog fleas. Already considered some of the most damaging alien animal species for global biodiversity, feral cats, foxes and rats are commonly observed to harbour flea infestations [17, 23, 24, 41, 42]. Previous authors have speculated on the role of feral hosts as reservoirs, suggesting that within its climatic limits, the cat flea is capable of using virtually any available feral mammalian host to sustain its population [15]. Feral species thrive at the human-wildlife interface [28, 31, 43], and we show that anthropogenic habitat use influence *C. felis* infestation risk. Collectively, our results suggest spatial overlap with feral reservoir hosts plays a crucial role in flea spillover to wildlife and will likely magnify spillover that is already driven by encroachment of flea-bearing domestic pets into natural habitats.

Our study adds to a growing body of empirical and theoretical evidence implicating invasive species as contributors of parasite spread [6, 44–46]. Yet in addition to feral mammals, urban-adapted native species may facilitate flea spillover. In the Americas, opossums (family Didelphidae) and raccoons (Procyonidae) are well-recognised as urban reservoirs for heavy cat flea infestations and flea-transmitted pathogens [17, 47–49]. Other urban-dwelling species such as European hedgehogs (*Erinaceus europaeus*; family Erinaceidae) have been found carrying cat fleas in Germany and Hungary [50, 51] as well as in New Zealand, where hedgehogs are introduced [52]. While this indicates increases in human footprints facilitate the spread of fleas, effects of urbanisation on parasite emergence are not well-understood. Some recent studies suggest parasites that commonly infect urban-adapted wildlife species exhibit increased prevalence in urban or suburban environments; while others find the opposite pattern [53, 54]. Fleas infesting domestic dogs, for example, will likely be more abundant in rural housing conditions where pets sleep on natural soils with high humidity, as opposed to urban housing that may be less suitable for nesting fleas [55, 56]. Studies that assess flea prevalence and infestation intensity across gradients of land use and domestic animal encroachment are needed to understand the true impacts of urbanisation on flea spillover.

Host switching and dispersal are key mechanisms underlying parasite spillover [57, 58]. While the geographic origins of cat and dog fleas are unknown [59], it is likely they spread to new regions following dispersal of humans and their pets [11]. This would have exposed fleas to a diversity of potential new host species. In our study, a strong signal of phylogeny for predicting host infestation suggests that conserved traits facilitated host switching following initial contact with reservoir hosts. Flea host range expansions may therefore follow a pattern of ‘ecological fitting’. This postulates that new host associations arise following contact with species that share traits with previous hosts [57, 60]. Ecological fitting has been observed in many host-parasite assemblages [60, 61]; however, uncovering the particular suite of conserved traits involved can be challenging. Our findings shed some light on the flea-mammal system, suggesting that body size and perhaps adaptability to urban environments are important for driving infestation risk. Broad similarities in habitat use and diet are either unimportant or too coarse to accurately identify patterns. Considering a wider array of traits that may influence flea exposure, such as nesting behaviour or local population density, would be useful to expand our understanding of spillover.

Ours is not the first study to suggest that cat fleas are more widespread, both in terms of geography and host-breadth, than dog fleas [50, 62, 63]. Many authors have speculated on why this occurs. Proposed hypotheses include a relatively restricted host range or restricted tolerance to extreme temperatures for the dog flea compared to the cat flea [15, 23]. While our data does not prove that infested hosts are maintaining flea populations, our findings highlight key differences in patterns of host use between the two flea species. Cat fleas are found on a much wider phylogenetic diversity of wild mammals than dog fleas. Host species that have been reported to carry dog fleas are restricted to certain phylogenetic clades, supporting hypothesis that dog fleas show higher host specificity than do cat fleas [10]. Experimental infestation studies, including co-infestations of *C. felis* with *C. canis*, coupled with additional field infestation data would be useful to rigorously test this hypothesis.

This study makes assumptions that any mammal species recorded to harbour a flea species has been searched for cat and dog fleas. This limitation that hinders our power to make predictions about infestation risk. On the flipside, there are likely many more confirmed associations between wild mammal hosts and fleas that our search methods failed to identify. Searching of Web of Science and PubMed could be extended to encompass mammal-flea associations for a broader range of flea species. This would serve to increase our understanding of flea biogeography while giving better resolution of host traits that influence risk of cat and dog flea infestation. We reinforce earlier calls for more detailed record-keeping to help identify informative processes involved in parasite spillover among wild host species [64, 65]. While the scope of our study was to collate data on flea-host associations and make inferences on species-level infestation risk, future studies addressing differences in infestation prevalence and intensity at the population-level would be informative for broadening our understanding of flea spillover.

## Conclusions

We find that cat fleas are among the most host-generalist of all ectoparasites, a trait that likely contributes to parasite spread at the human-wildlife interface. We suggest that reducing wild species’ contact rates with domestic animals across natural and anthropogenic habitats, together with mitigating impacts of invasive reservoir hosts, will be crucial for reducing invasive flea infestations in wild mammals. Crucial to developing management strategies will be differentiating between incidental hosts and those capable of maintaining and spreading fleas throughout the parasite lifecycle.

## Methods

### Compiling a global flea host-parasite database

We searched PubMed (National Library of Medicine National Institutes of Health, US) and Web of Science (Clarivate Analytics, US) to identify publications that describe cat and/or dog flea infestations in free roaming wild and domestic species. These databases apply hierarchical search algorithms to cover a broad range of nested terms; for instance, searching ‘ruminant’ will also search terms nested within ruminant, such as ‘goat’, ‘cattle’ etc. (Additional file 2 for details of literature accession methods and specific search terms). From identified papers, we recorded host species, presence/absence of *C. felis* and *C. canis*, and, if data on individuals sampled was available, number of hosts sampled and number infested with each flea species. Fleas regarded as *Ctenocephalides* spp. (i.e. only identified to genus level) were recorded as unidentified *Ctenocephalides* species. Note that few studies distinguished between *C. felis* subspecies, and so all *C. felis* records were grouped as a single category. Further flea host-parasite records were gathered from the Global Mammal Parasite Database v2.0 [66] and the Natural History Museum Database, London, UK (http://www.nhm.ac.uk/research-curation/scientific-resources/biodiversity/uk-biodiversity/british-flea-distribution/; accessed 06/06/17).

To make inferences about traits that best predict the probability that wildlife are infested with either flea species, we gathered a list of all wild mammal species known to have been sampled for fleas. We included hosts from published flea host-parasite community datasets ([67] from Palaearctic regions; [68] from Serbia) and comprehensive flea-host checklists. We also included mammal species that have been recorded to harbour arthropod ectoparasites in the Global Mammal Parasite Database v2.0 [66]. For all mammal species included, associations with cat and dog fleas were recorded as binary variables (present or absent). To account for possible sampling bias among species, we queried the number of published references for each binomial species name from the Scopus literature database (https://www.scopus.com; accessed 08/06/17) using accompanying search terms ‘parasite’ and ‘ectoparasite’. We are aware that our list of host species is incomplete, but we believe our database is sufficiently representative to explore variable wildlife traits that may influence likelihoods of cat and dog flea infestation. The final database included 446 unique host-parasite-location observations.

### Mammalian host phylogeny and ecological trait data

For all sampled mammal species, we gathered ecological trait data from the International Union for Conservation of Nature (IUCN; http://www.iucnredlist.org/; accessed 04/05/17), EltonTraits 1.0 [69], PanTheria [70] and habitat diversity [71] databases to include attributes likely to distinguish hosts in terms of availability and suitability for flea infestations. Selected traits included: body mass, linked to longevity and adaptation to environments; diet diversity (a Shannon index based on species’ proportional use of 10 diet categories represented in EltonTraits); habitat use (binary indicators of whether a species uses each of 18 IUCN habitat categories); cohabitation diversity (a co-occurrence ß diversity metric quantifying the target species’ degree of habitat and community specialization, where a ‘generalist’ occurs in a range of habitats that differ in species composition while a ‘specialist’ uses habitats that contain a consistent collection of other mammal species; [71]); IUCN threat status; mid-range latitude; and mid-range longitude. To test for differences among specific habitat types in logistic regressions, IUCN habitat variables were used to create binary indicators that reflect whether species use anthropogenic (‘introduced vegetation’ or ‘artificial terrestrial’), forest (‘forest’ or ‘shrubland’) and dry bush habitats (‘desert’, ‘savanna’ or ‘grassland’). Species’ phylogenetic relationships were estimated from a recent mammalian supertree [72].

### Phylogenetic logistic regressions

Infestation probability for each flea species was modelled separately using species-level infestation data of all mammal species with one of the two focal flea species as the response (‘1’ if a species has been recorded as infested; ‘0’ if a species has not been recorded as infested). We tested whether host attributes influence infestation probability using a hierarchical logistic regression with a logit link function. Predictor variables included host body mass, diet diversity, cohabitation diversity, anthropogenic habitat use, forest habitat use, dry bush habitat use, citation counts linked to ‘parasite’ and citation counts linked to ‘ectoparasite’. We included an interaction between cohabitation diversity and anthropogenic habitat use to test if species that rely more on anthropogenic habitats have increased infestation risk (where species that use anthropogenic habitats and have low cohabitation diversity indices are assumed to rely more heavily on man-made habitats than those with higher diversity indices). To account for underlying structure driven by host phylogenetic relationships or recent population trends, host phylogeny and IUCN threat status were included as random grouping terms, allowing inferences for group-specific slopes whilst estimating between-group variation [73].

The model was fitted in a Bayesian framework with Markov Chain Monte Carlo (MCMC) sampling using the R package *MCMCglmm* [74]. We used parameter expansion (redundant multiplicative reparameterisation of the linear model) for the threat status variance component to reduce dependence among parameters and improve chain mixing [75]. For the phylogenetic variance component, we used a *χ*^2^ distribution with one degree of freedom, which improves sampling properties and heritability estimates for binary outcomes [75, 76]. Residual variance was fixed at 1, as this variance is non-identifiable when estimating binary outcomes [76]. All continuous predictors were centred and scaled (dividing by one SD) prior to regression. We ran two chains for 2000,000 iterations each, removing 1000,000 as ‘burn-in’ and with a thinning value of 1000 (2000 total posterior samples for each parameter). Chain mixing was inspected visually and with the Gelman-Rubin diagnostic (all values < 1.2). Autocorrelations were calculated to ensure independence of consecutive samples (all autocorrelations < 0.1). Because a limited number of records in our database included number of hosts sampled and number infested (e.g. 78 observations from 33 host species for *C. felis*), power to detect prevalence patterns was low and we focused only on species-level associations.

### Parasite functional-phylogenetic host specificity

We calculated observed and expected host specificity for each flea species to assess whether fleas use hosts that are more similar based on phylogeny, habitat use or diet than expected by chance. We used the functional-phylogenetic host specificity metric described by Clark & Clegg [77], which integrates host phylogenetic and ecological distances to quantify their relative influences on parasite host specificity.

To describe similarity between host habitat and diet niches, we applied hierarchical clustering to dissimilarity Gower’s distance matrices [78]; the first matrix incorporated host micro-habitat traits (9 terrestrial habitat use binary indicator variables) and macro-habitat traits (co-occurrence diversity; midrange longitude and midrange latitude; all as continuous variables). The second matrix incorporated two host diet traits (a fuzzy variable to describe the proportional use 10 diet categories and the Shannon diet diversity continuous variable). All continuous variables were scaled by one SD and weighted by the inverse of their phylogenetic autocorrelations to capture variance in niches not captured by phylogeny. We used Abouheif’s C, a metric efficient at detecting phylogenetic autocorrelation regardless of topology [79, 80]. Distance matrices were built using weighted variables following Pavoine et al. [81]. Uncertainty in host-parasite analyses is important to incorporate when assessing host-specificity and infestation risk [3, 82]. Because different hierarchical clustering algorithms lead to different inferences [83], we generated eight dendrogram topologies from each matrix (habitat and diet) to capture uncertainty in relationships.

Phylogenetic and dendrogram branch lengths were scaled (dividing distances by the maximum distance for each tree) so pairwise distances ranged from zero to one. Pairwise phylogenetic and niche distances (*PDist* and *FDist*, respectively) were then used to calculate functional-phylogenetic distance (*FPDist*):

1 $$ FPDist={\left(a{PDist}^p\kern0.5em +\kern0.5em \left(1\kern0.5em -\kern0.5em a\right){FDist}^p\right)}^{1/p\operatorname{}} $$

The weighting parameter *α* varies from zero to one; values approaching one give greater weight to *PDist*; *a* values approaching zero give greater weight to *FDist*. We set *p* = 2 to calculate squared Euclidean *FPDist* distances. Host *FPDist* distances were used to calculate a phylospecificity index (STD*) for each parasite, using species-level infestation data and following Clark & Clegg [77]. Null host distributions were created for each parasite by randomly drawing the observed number of infested species from the sampled host pool and calculating expected STD*. We allowed *α* to vary across a uniform distribution from zero to one to alter relative weights of host phylogeny and ecological niche in each draw. Expected STD* values were subtracted from observed to yield specificity differentials. These will be negative if hosts are more similar than expected (clustered) and positive if hosts are less similar (overdispersed). This was repeated 10,000 times to generate distributions of STD* differentials for each flea species. Separate analyses were conducted using host habitat and diet niche dendrograms. To allow for comparisons between flea species and account for uncertainty in the large number of *C. felis* host-parasite records (138 host species, see Results), we randomly selected 31 observed host species for *C. felis* analyses in each draw (equal to the number of host species observed to carry *C. canis*; see Results).

For all analyses, we report posterior modes and 95% credible intervals (highest posterior density intervals for logistic regressions; 2.5% and 97.5% quantiles for host specificity indices). Effects were considered ‘significant’ if credible intervals did not include zero. Of the 685 sampled mammal species (see Results), we were able to collate trait data for 639 species. These 639 species were included in logistic regressions (MCMCglmm cannot impute missing predictors), while the full set of 685 species was used for host specificity analyses (missing trait data was imputed from the full range of observed values for each trait).

## Additional files

Additional file 1: Figure S1. Regression coefficients for fixed effects included in the logistic regressions to predict flea infestation probability in wild mammal species. Markers indicate posterior modes and line segments represent 95% highest posterior density credible intervals (CIs). Terms considered significant (95% CIs do not include zero) are highlighted in red. (DOCX 58 kb)

Additional file 2: Overview of literature search methods. This file presents an overview of the methods used to search literature and scrape flea*host checklists. R code for performing the systematic search and scraping online checklists can be found on figshare (https://doi.org/10.6084/m9.figshare.5623705.v2). (DOCX 15 kb)
