# Supplementary material for: Parasite spread at the domestic animal - wildlife interface: anthropogenic habitat use, phylogeny and body mass drive risk of cat and dog flea (Ctenocephalides spp.) infestation in wild mammals
Source: Parasit Vectors. 2018 Jan 8;11:8. doi: 10.1186/s13071-017-2564-z (PMC5757300; doi:10.1186/s13071-017-2564-z)
Supplement: Supplementary file 1 — Regression coefficients for fixed effects included in the logistic regressions to predict flea infestation probability in wild mammal species. Markers indicate posterior modes and line segments represent 95% highest posterior density credible intervals (CIs). Terms considered significant (95% CIs do not include zero) are highlighted in red. (DOCX 58 kb) [file 13071_2017_2564_MOESM1_ESM.docx]

**Additional File 1: Figure S1**

**Parasite spread at the domestic animal - wildlife interface: anthropogenic habitat use, phylogeny and body mass drive risk of cat and dog flea (*Ctenocephalides* spp.) infestation in wild mammals**

Nicholas J Clark^1^*, Jennifer Seddon^1^, Jan Šlapeta^2^ and Konstans Wells^3^

^1^School of Veterinary Science, University of Queensland, Gatton Queensland 4343, Australia

^2^Sydney School of Veterinary Science, Faculty of Science, University of Sydney, Sydney New South Wales 2006, Australia

^3^Environmental Futures Research Institute, Griffith University, Nathan Queensland 4111, Australia

*Correspondence: nicholas.j.clark1214@gmail.com

Author email addresses:

J. Seddon: j.seddon1@uq.edu.au

J. Šlapeta: jan.slapeta@sydney.edu.au

K. Wells: konswells@gmail.com


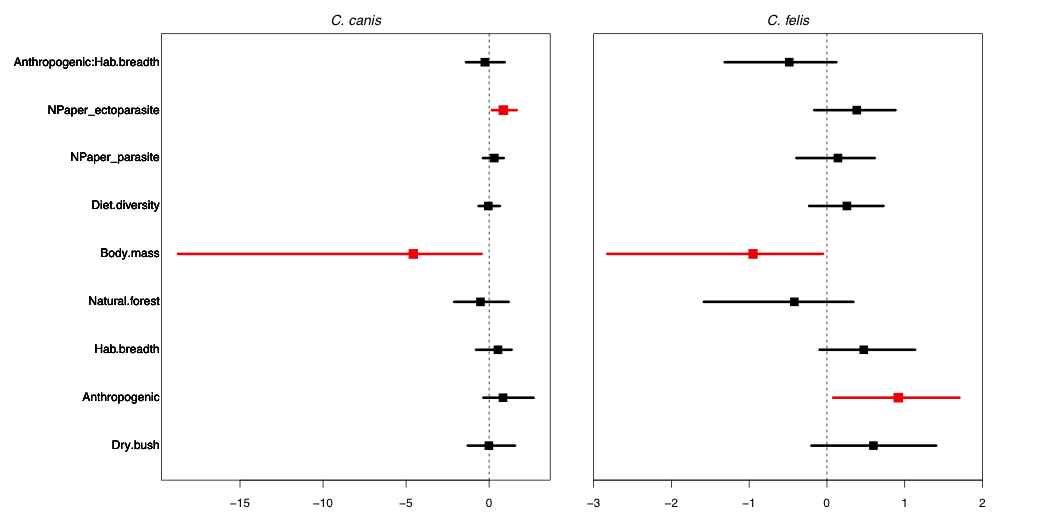


**Figure S1.** Regression coefficients for fixed effects included in the logistic regressions to predict flea infestation probability in wild mammal species. Markers indicate posterior modes and line segments represent 95% highest posterior density credible intervals (CIs). Terms considered significant (95% CIs do not include zero) are highlighted in red.
