# Supplementary material for: Parasite spread at the domestic animal - wildlife interface: anthropogenic habitat use, phylogeny and body mass drive risk of cat and dog flea (Ctenocephalides spp.) infestation in wild mammals
Source: Parasit Vectors. 2018 Jan 8;11:8. doi: 10.1186/s13071-017-2564-z (PMC5757300; doi:10.1186/s13071-017-2564-z)
Supplement: Supplementary file 2 — Overview of literature search methods. This file presents an overview of the methods used to search literature and scrape flea*host checklists. R code for performing the systematic search and scraping online checklists can be found on figshare (https://doi.org/10.6084/m9.figshare.5623705.v2). (DOCX 15 kb) [file 13071_2017_2564_MOESM2_ESM.docx]

**Additional File 2: Overview of literature search methods**

**Parasite spread at the domestic animal - wildlife interface: anthropogenic habitat use, phylogeny and body mass drive risk of cat and dog flea (*Ctenocephalides* spp.) infestation in wild mammals**

Nicholas J Clark^1^*, Jennifer Seddon^1^, Jan Šlapeta^2^ and Konstans Wells^3^

^1^School of Veterinary Science, University of Queensland, Gatton Queensland 4343, Australia

^2^Sydney School of Veterinary Science, Faculty of Science, University of Sydney, Sydney New South Wales 2006, Australia

^3^Environmental Futures Research Institute, Griffith University, Nathan Queensland 4111, Australia

*Correspondence: nicholas.j.clark1214@gmail.com

Author email addresses:

J. Seddon: j.seddon1@uq.edu.au

J. Šlapeta: jan.slapeta@sydney.edu.au

K. Wells: konswells@gmail.com

This file presents an overview of the methods used to search literature and scrape flea*host checklists. R code for performing the systematic search and scraping online checklists can be found on figshare (https://doi.org/10.6084/m9.figshare.5623705.v2).

**Step 1: Gathering abstracts to search for flea infestations of wildlife around the globe, using PubMed** ***(searching with the ‘rentrez’ R package)***

1. Terms: “((cat flea OR ctenocephalides) AND (rodent OR rat OR murine))”

2. Terms: “((cat flea OR ctenocephalides) AND (wildlife OR wild OR free-roaming OR free roaming))”

3. Terms: “((cat flea OR ctenocephalides) AND (bird OR avian OR chicken))”

4. Terms: “((cat flea OR ctenocephalides) AND (rabbit OR fox OR deer OR goat OR pig OR bear OR livestock OR cattle OR ruminant OR feral))”

**Total: 245 papers**

**Step 2: Keeping or discarding collected papers by title and abstract**

We manually examined titles and abstracts of all papers, keeping those that examine wild animals, free-roaming feral animals (including farm animals and ‘domestic’ animals in villages such as Borneo and Laos). We discarded papers that only examine house-kept domestic pets, humans or that were laboratory-based, or that only focused on non-*Ctenocephalides* (either *canis* or *felis*) fleas.

**New Total: 122 papers**

**Step 3: Repeat searches in Web of Science and compare to the Pubmed list**

We searched Web of Science using identical terms in ‘topic’ field, downloading results as a tab-delimited text file then importing into R to compare to the specified PubMed list (based on PubMed identification, title and abstract) to find unique records.

**New Total: 506 papers**

**Step 5: Repeat manual screening on the new subset of papers**

**New Total: 181 papers**

**Step 6: Check for additional records from checklists and informative websites (search references and citations of checklists)**

<http://www.cvbd.org/en/flea-borne-diseases/about-fleas/general-aspects/origin-and-distribution/>

<http://fleascience.com/flea-encyclopedia/flea-infestations/are-cat-and-dog-fleas-the-same/#zp-ID-12738-1543244-BMQ6ZVP4>

**Step 7: Search the Global Mammalian Parasite Database v2.0 (Stephens et al. 2017) and British Natural History Museum flea collection (http://www.nhm.ac.uk/research-curation/scientific-resources/biodiversity/uk-biodiversity/british-flea-distribution/database/Fleas.do) for any additional host-parasite records**

**Methods for gathering lists of mammal species sampled for flea infestations**

1) Included all host species in the Global Mammal Parasite Database that have been recorded to harbour arthropod ectoparasites (assuming that these host species were searched for any external arthropod parasites)

2) Included all host species from published flea host-parasite community datasets (Hadfield et al. 2013 from Palearctic regions; Pilosof et al. 2017 from Serbia)

3) Scraped all species binomial names from all pages of the online checklist of flea*host interactions in South Africa (Segerman)

4) Manually copied and parsed text for all host binomials from flea host*parasite checklists from the USA (New Mexico,Tennessee and South Carolina), Canada (THE FLEAS OF CANADA, ALASKA AND GREENLAND (SIPHONAPTERA)), Australia (A Monograph of Australian Fleas (Siphonaptera); CSIRO) and Britain (Handbook of British Fleas (http://www.nhm.ac.uk/research-curation/scientific-resources/biodiversity/uk-biodiversity/british-flea-distribution/index.html)

5) Searched all collected binomials and other texts that could represent mammalian hosts (i.e. ‘C. lupus’ or text parsing errors such as ‘Canis lumus’) using pattern matching against IUCN taxonomy (function tax_name in the *taxize* package). Kept all species binomials that are found to belong to class Mammalia

6) For remaining text that doesn't match to a record in IUCN (including many errors in text parsing), used incomplete pattern matching against mammalian IUCN taxonomy (function *agrep* with max.distance = 0.15). Returned all possible matches and then manually selected the most likely names.

**References**

Stephens PR, Pappalardo P, Huang S, Byers JE, Farrell MJ, Gehman A, et al. Global Mammal Parasite Database version 2.0. Ecology. 2017;98(5):1476-1476.

Hadfield JD, Krasnov BR, Poulin R, Nakagawa S. A tale of two phylogenies: comparative analyses of ecological interactions. Am Nat. 2013;183(2):174-187.

Pilosof S, Fortuna MA, Vinarski MV, Korallo‐Vinarskaya NP, Krasnov BR. Temporal dynamics of direct reciprocal and indirect effects in a host-parasite network. J Anim Ecol. 2013;82(5):987-996.
